# Recent trends and patterns in HIV‐1 transmitted drug resistance in the United Kingdom

**DOI:** 10.1111/hiv.12414

**Published:** 2016-08-01

**Authors:** A Tostevin, E White, D Dunn, S Croxford, V Delpech, I Williams, D Asboe, A Pozniak, D Churchill, AM Geretti, D Pillay, C Sabin, A Leigh‐Brown, E Smit, the UK HIV Drug Resistance Database, Celia Aitken, Patricia Cane, David Chadwick, Duncan Clark, Simon Collins, Samuel Douthwaite, Esther Fearnhill, Kholoud Porter, Christophe Fraser, Antony Hale, Stéphane Hué, Steve Kaye, Paul Kellam, Linda Lazarus, Tamyo Mbisa, Nicola Mackie, Samuel Moses, Chloe Orkin, Eleni Nastouli, Andrew Phillips, Kate Templeton, Peter Tilston, Hongyi Zhang, Keith Fairbrother, Jane Greatorex, Siobhan O'Shea, Jane Mullen, Alison Cox, Richard Tandy, Tracy Fawcett, Mark Hopkins, Lynne Ashton, Clare Booth, Ana Garcia‐Diaz, Jill Shepherd, Matthias L. Schmid, Brendan Payne, Spiro Pereira, Jonathan Hubb, Stuart Kirk, Rory Gunson, Amanda Bradley‐Stewart

**Affiliations:** ^1^ MRC Clinical Trials Unit at UCLUniversity College LondonLondonUK; ^2^ Centre for Infectious Disease Surveillance and Control (CIDSC)Public Health EnglandLondonUK; ^3^ Mortimer Market CentreUniversity College London Hospitals NHS TrustLondonUK; ^4^ Chelsea & Westminster HospitalLondonUK; ^5^ Brighton and Sussex University Hospitals NHS TrustBrightonUK; ^6^ University of LiverpoolLiverpoolUK; ^7^ Division of Infection and ImmunityUniversity College LondonLondonUK; ^8^ Africa Centre for Health and Population StudiesUniversity of KwaZulu‐NatalMtubatubaSouth Africa; ^9^ Research Department of Infection and Population HealthUniversity College LondonLondonUK; ^10^ University of EdinburghEdinburghUK; ^11^ Public Health EnglandBirmingham Heartlands HospitalBirminghamUK

**Keywords:** drug resistance, HIV‐1, mutations, transmitted, transmitted drug resistance, transmitted drug resistance mutations

## Abstract

**Objectives:**

Transmission of drug‐resistant HIV‐1 has decreased in the UK since the early 2000s. This analysis reports recent trends and characteristics of transmitted drug resistance (TDR) in the UK from 2010 to 2013.

**Methods:**

Resistance tests conducted in antiretroviral treatment (ART)‐naïve individuals between 2010 and 2013 were analysed for the presence of transmitted drug resistance mutations (TDRMs), defined as any mutations from a modified 2009 World Health Organization surveillance list, or a modified 2013 International Antiviral Society‐USA list for integrase tests. Logistic regression was used to examine associations between demographics and the prevalence of TDRMs.

**Results:**

TDRMs were observed in 1223 (7.5%) of 16 425 individuals; prevalence declined from 8.1% in 2010 to 6.6% in 2013 (*P* = 0.02). The prevalence of TDRMs was higher among men who have sex with men (MSM) compared with heterosexual men and women (8.7% versus 6.4%, respectively) with a trend for decreasing TDRMs among MSM (*P* = 0.008) driven by a reduction in nucleoside reverse transcriptase inhibitor (NRTI)‐related mutations. The most frequently detected TDRMs were K103N (2.2%), T215 revertants (1.6%), M41L (0.9%) and L90M (0.7%). Predicted phenotypic resistance to first‐line ART was highest to the nonnucleoside reverse transcriptase inhibitors (NNRTIs) rilpivirine and efavirenz (6.2% and 3.4%, respectively) but minimal to NRTIs, including tenofovir, and protease inhibitors (PIs). No major integrase TDRMs were detected among 101 individuals tested while ART‐naïve.

**Conclusions:**

We observed a decrease in TDRMs in recent years. However, this was confined to the MSM population and rates remained stable in those with heterosexually acquired HIV infection. Resistance to currently recommended first‐line ART, including integrase inhibitors, remained reassuringly low.

## Introduction

The majority of individuals initiating combination antiretroviral treatment (ART) suppress virus replication and are less likely to acquire drug resistance compared with individuals who were exposed to older ART regimens. However, approximately one‐third of individuals in the UK experiencing virological failure still have drug‐resistant strains of HIV 1. Onward transmission of drug‐resistant HIV can have an adverse effect on the success of first‐line treatment and may negatively impact an individual's prognosis 2. National guidelines in the UK 3 and many other European countries 4 recommend that newly diagnosed individuals have a resistance test to detect transmitted drug resistance (TDR) and help selection of a fully active first‐line treatment regimen.

Surveillance of TDR in the UK has monitored the impact of improved ART regimens and resistance testing guidelines over time. Previous work reported a decrease in the prevalence of TDR between 2001 and 2007 5, 6. The prevalence of TDR in individuals with a subtype B infection was higher than in those with non‐B infections between 2001 and 2006, reflecting long‐standing free access to ART in the UK and limited availability of ART in countries where non‐B infections were probably acquired 5. The prevalence of TDR in individuals with a subtype B infection increased slightly between 2007 and 2009, driven by an increase in resistance to the nucleoside reverse transcriptase inhibitor (NRTI) drug class, leading to the hypothesis that onward transmission of persistent thymidine analogue mutations (TAMs) among undiagnosed men who have sex with men (MSM) may keep levels of TDR stable in this population 6.

The European SPREAD. collaborative study reported a similar pattern of a higher prevalence of TDR among MSM compared with individuals who acquired their infection heterosexually between 2002 and 2007 7. Other studies in high‐income countries have reported comparable rates of TDR; the European CHAIN collaboration reported a prevalence of 9.5% between 1998 and 2008 2, while a prevalence of 15% was reported in a US study conducted between 1999 and 2011 8.

Of increasing interest in recent years is the surveillance of TDR to integrase inhibitors, a relatively new drug class approved for use in HIV treatment in Europe in 2008. The integrase inhibitors raltegravir, dolutegravir and elvitegravir are increasingly likely to be used as first‐line ART in combination with two NRTIs. Other than a small number of isolated case reports 9, 10, 11, studies have failed to detect transmitted major integrase mutations [as defined by the International Antiviral Society (IAS)‐USA 2013 list 12] in ART‐naïve individuals 13, 14, 15. Recently, the French PRIMO cohort study detected isolated E157Q mutations in 1.5% of baseline samples 16.

The objective of this report was to examine the recent prevalence, patterns and predictive factors of TDR among ART‐naïve persons living with diagnosed HIV infection in the UK.

## Methods

The UK HIV Drug Resistance Database (UK HDRD) collects information from all National Health Service and Public Heath England (PHE) virology laboratories performing resistance tests as part of routine clinical care in the UK. Nucleotide sequences of the protease and reverse transcriptase and integrase genes are collected. Resistance data are linked to demographic and clinical patient data held in the UK Collaborative HIV Cohort study (UK CHIC) database 17 and the national HIV/AIDS Reporting System (HARS) database held at PHE 18. Eighty‐three per cent of resistance tests are successfully linked to one or both sources.

Resistance testing of polymerase (*pol*) (protease and reverse transcriptase genes) started in the UK in 1998, but testing of ART‐naïve individuals only became widespread from 2005 following the release of British HIV Association (BHIVA) guidelines recommending routine monitoring of transmitted resistance in this population 3. Sequencing of the integrase gene was first performed in the UK in 2007 but to date only a small number of integrase sequences have been reported from ART‐naïve individuals. Findings in the *pol* and integrase genes are described separately in this report. We selected the first sequence per individual aged 15 years or over who had not yet received ART at the time of sampling. Individuals infected via mother‐to‐child transmission were excluded.

In this report, the term ‘transmitted drug resistance mutations’ (TDRMs) is used to describe (a) for *pol*, the presence of one or more mutations from the World Health Organisation (WHO) 2009 surveillance list 19 with the addition of E138K, a mutation known to cause resistance to the second‐generation nonnucleoside reverse transcriptase inhibitor (NNRTI) rilpivirine, and all changes at codon T215 as they are likely to be ancestrally related to T215F or Y mutations 20; (b) for integrase, the presence of mutations from the IAS‐USA 2013 list or accessory mutations reported by the Stanford HIVdb algorithm v7.0 (HIV Drug Resistance Database, Stanford University, Stanford, CA). HIV‐1 subtypes were determined using the rega v3.0 genotyping tool (REGA Institute, Katholieke Universiteit Leuven, Belgium). Predicted phenotypic resistance to ART drugs currently recommended (preferred or alternative) for first‐line treatment of HIV infection in the UK 21 was examined using the Stanford HIVdb algorithm v7.0. Scores of low‐level, intermediate or high‐level resistance were used to predict phenotypic resistance.

Univariate and multivariate logistic regression was used to examine the association between demographic and clinical factors and the prevalence of TDRMs. Demographic and clinical factors included in logistic regression models were: year of resistance test, transmission group, ethnicity, age at diagnosis, region of treatment centre and baseline CD4 T‐cell count and HIV‐1 viral load. Recent Infection Testing Algorithm (RITA) avidity score was not included in multivariate analysis given the close relationship to baseline CD4 cell count and the availability of more CD4 data. Viral subtype was not included as a separate variable in either the univariate or multivariate analyses because of the close association with ethnicity and transmission group. Where demographic information was missing, an unknown category was created to allow individuals to be included in multivariate analyses. *A priori*, an interaction between year of resistance test and transmission group was added to the adjusted model. Wald tests were used to assess the effect of demographic factors on the prevalence of TDRMs in the adjusted model. Individuals were defined as being recently infected when this was confirmed with a RITA avidity score <80% and their first resistance test was within 3 months of HIV diagnosis. The closest CD4 cell count and HIV‐1 viral load measured within the year before or the 6 months after the resistance test and before receiving ART were included in analyses.

Analyses of time trends of TDRMs in *pol* are presented from 2005 onwards to provide contextual data for recent years (2010‐2013), which are the focus of the report. More detailed analyses of predicted phenotypic resistance and predictors of TDRMs are limited to 2010‐2013 to be most relevant to contemporary clinical practice. Data on TDRMs conferring resistance to integrase inhibitors cover the period 2007 to 2013.

All analyses were carried out using stata statistical software version 13.0 (StataCorp LP, College Station, TX, USA).

## Results

A total of 40 549 individuals were tested for TDRMs (*pol*) while ART‐naïve between 2005 and 2013 (Table 1). Of the 16 425 individuals who were tested in recent years (2010–2013 inclusive), 66.5% were male and 49.7% of white ethnicity. MSM comprised 45.9% of individuals, with 39.4% acquiring their infection heterosexually. Median age at HIV diagnosis in recent years was 35 years [interquartile range (IQR) 28–44 years]. Almost half of all samples (45.6%) came from treatment centres in London, followed by 19.5% from the Midlands and East England region. Median CD4 cell count at the time of the resistance test was 350 cells/mL (IQR 175–530 cells/μL). As expected, the median CD4 cell count was higher in those with a recent infection confirmed by a RITA assay: 546 cells/μL (1098 individuals) compared with 305 cells/μL (4864 individuals) in nonrecent infections. The median viral load at the time of the resistance test was 4.76 log_10_ HIV‐1 RNA copies/mL (IQR 4.12–5.31 copies/mL). The median time from HIV diagnosis to first resistance test was 9 days. Subtype B viruses were the most commonly detected (49.2%), followed by subtype C (21.7%), subtype CRF02_AG (7.0%) and subtype A (5.8%). Only 16.0% of heterosexual men and women were infected with subtype B virus, while 42.6% had subtype C infection. Conversely, almost 80% of MSM had subtype B virus and only 3.5% subtype C. Demographic characteristics of ART‐naïve individuals tested for drug resistance in recent years were comparable to those of individuals newly diagnosed with HIV infection in the UK between 2010 and 2013 22, suggesting that our cohort is broadly representative of this population in the UK.

**Table 1:** Demographic and clinical patient characteristics, 2005–2013

Patient characteristics	Year of resistance test		Year of HIV diagnosis
	2005–2009	2010–2013	2010–2013^a^
Total *n*	24 124	16 425	24 828
Sex			
Male	16050 (66.5)	10917 (66.5)	17769 (71.6)
Female	7133 (29.6)	3820 (23.3)	7059 (28.4)
Unknown	1941 (8)	1688 (10.3)	0
Transmission group			
MSM	10057 (41.7)	7533 (45.9)	11678 (47.0)
Heterosexual male	4045 (16.8)	2833 (17.2)	4540 (18.3)
Heterosexual female	6833 (28.3)	3635 (22.1)	6119 (24.6)
IDU	465 (1.9)	298 (1.8)	530 (2.1)
Other	642 (2.7)	125 (0.8)	506 (2.0)
Unknown	2082 (8.6)	2001 (12.2)	1455 (5.9)
Ethnicity			
White	11361 (47.1)	8169 (49.7)	13222 (53.3)
Black African	7792 (32.3)	3932 (23.9)	6741 (27.2)
Black Caribbean	830 (3.4)	565 (3.4)	708 (2.9)
Asian	621 (2.6)	720 (4.4)	1219 (4.9)
Other	1529 (6.3)	1139 (6.9)	1772 (7.1)
Unknown	1991 (8.3)	1900 (11.6)	1166 (4.7)
Age at diagnosis (years)			
[median (IQR)]	34 (28–41)	35 (28–44)	36 (29–45)
Region of treatment centre/diagnosis			
London	11557 (47.9)	7493 (45.6)	10823 (43.6)
South England	2811 (11.7)	2175 (13.2)	3542 (14.3)
North England	3778 (15.7)	2237 (13.6)	3997 (16.1)
Midlands and East England	3638 (15.1)	3208 (19.5)	4370 (17.6)
Wales	497 (2.1)	441 (2.7)	562 (2.3)
Scotland	1316 (5.5)	435 (2.6)	1135 (4.6)
Northern Ireland	198 (0.8)	252 (1.5)	358 (1.4)
Other/unknown	329 (1.4)	184 (1.1)	41 (0.2)
Baseline CD4 count			
<200 cells/mL	4935 (20.5)	3249 (19.8)	5557 (22.4)
200–349 cells/mL	3909 (16.2)	2508 (15.3)	4259 (17.2)
350–499 cells/mL	3353 (13.9)	2528 (15.4)	4541 (18.3)
≥500 cells/mL	4032 (16.7)	3342 (20.3)	6728 (27.1)
Unknown	7895 (32.7)	4798 (29.2)	3743 (15.1)
CD4 count (cells/mL) [median (IQR)]	320 (160–497)	350 (175–530)	371 (187–558)
Baseline VL			
<4.0 log_10_ copies/mL	2579 (10.7)	1262 (7.7)	–
4.0 to <5.0 log_10_ copies/mL	3833 (15.9)	2396 (14.6)	–
≥5.0 log_10_ copies/mL	2659 (11)	2268 (13.8)	–
Unknown	15053 (62.4)	10499 (63.9)	–
VL [median (IQR)]	4.54 (3.91–5.11)	4.76 (4.13–5.31)	–
Subtype			
A	1364 (5.7)	957 (5.8)	–
B	11761 (48.8)	8085 (49.2)	–
C	6742 (27.9)	3557 (21.7)	–
G	586 (2.4)	438 (2.7)	–
CRF01_AE	587 (2.4)	560 (3.4)	–
CRF02_AG	1324 (5.5)	1150 (7)	–
Other	1760 (7.3)	1678 (10.2)	–
Time from diagnosis to resistance test (days) [median (IQR)])	19 (4–239)	9 (1–36)	–

IDU, injecting drug use; IQR, interquartile range; MSM, men who have sex with men; VL, viral load.

Values are *n* (%), unless otherwise stated.

a Data from PHE HIV surveillance data tables 2015 (https://www.gov.uk/government/statistics/hiv-data-tables) 22.

**Figure 1:**
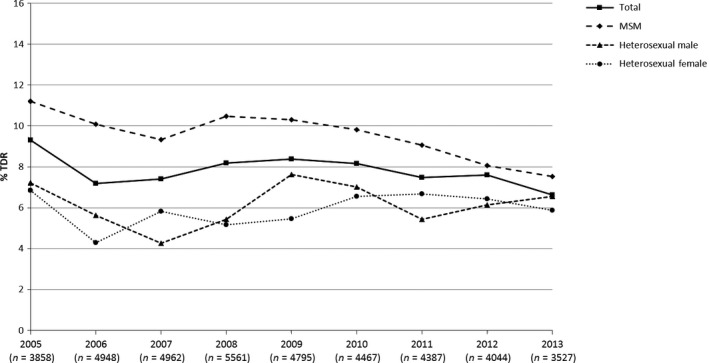
Prevalence of transmitted drug resistance mutations (TDRMs) by transmission group, 2005−2013. MSM, men who have sex with men.

The prevalence of TDRMs has declined over time, from 9.3% in 2005 to 6.6% in 2013 (test for trend *P* = 0.02) (Fig. 1). As reported previously, the rate stabilized between 2008 and 2010, but has declined in recent years from 8.1% in 2010 to 6.6% in 2013 (test for trend *P* = 0.02). This recent decline was driven by a decrease in the prevalence of observed TDRMs in MSM (9.8% in 2010 to 7.5% in 2013; test for trend *P* = 0.008), while the prevalence of TDRMs in heterosexual men and women has remained stable in recent years (6.7% in 2010 and 6.2% in 2013; test for trend *P* = 0.56). The decrease in observed TDRMs in MSM was driven by a reduction in the prevalence of TDRMs conferring resistance to the NRTI drug class (from 5.2% in 2010 to 3.7% in 2013; test for trend *P* = 0.01), while the prevalence of TDRMs conferring resistance to NNRTIs and protease inhibitors (PIs) was unchanged over the 4 years (test for trend *P* = 0.63 for NNRTIs; *P* = 0.12 for PIs).

Further analysis of individuals tested between 2010 and 2013 revealed that, of the 1233 (7.5%) individuals with one or more resistance mutations, 6.5% had resistance to a single drug class, 0.9% had dual drug class resistance and 0.1% had triple drug class resistance (Table 2). Overall, 3.5% had TDRMs conferring resistance to drugs from the NRTI class, 3.3% to the NNRTI class and 1.7% to the PI drug class. The most frequently detected mutations were T215 revertants (not F/Y) (268; 1.6%) and other TAMs [M41L (141; 0.9%) and K219Q/N (104; 0.6%)] conferring resistance to the NRTI drug class; K103N (354; 2.2%) and Y181C (66; 0.4%) conferring resistance to the NNRTI drug class, and L90M (111; 0.7%) and M46L (48; 0.3%) conferring resistance to the PI drug class. Despite TAMs remaining the most commonly detected NRTI resistance mutations, their prevalence has been decreasing in recent years, from 4.5% in 2010 to 3.1% in 2013 (test for trend *P* = 0.001). K65R was only detected in 10 (0.06%) individuals tested between 2010 and 2013.

**Table 2:** Transmitted drug resistance mutations (TDRMs) by drug class, 2010–2013

TDRMs by drug class	*n*	%
Any	1233	7.5
Single		
NRTI only	425	2.6
NNRTI only	405	2.5
PI only	241	1.5
Dual		
NRTI + NNRTI	121	0.7
NRTI + PI	17	0.1
NNRTI + PI	11	0.1
Triple		
NRTI + NNRTI + PI	13	0.1

NRTI, nucleoside reverse transcriptase inhibitor; NNRTI, nonnucleoside reverse transcriptase inhibitor; PI, protease inhibitor.

**Figure 2:**
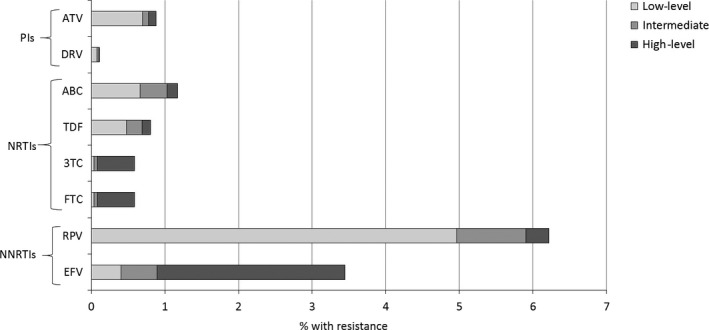
Predicted phenotypic resistance (Stanford scores) for antiretroviral drugs currently recommended for first‐line combination therapy in the UK, 2010−2013. 3TC, lamivudine; ABC, abacavir; ATV, atazanavir; DRV, darunavir; EFV, efavirenz; FTC, emtricitabine; PI, protease inhibitor; NRTI, nucleoside reverse transcriptase inhibitor; NNRTI, nonnucleoside reverse transcriptase inhibitor; RPV, rilpivirine; TDF, tenofovir.

Table 3 shows a comparison between the prevalence of TDRMs by drug class and predicted phenotypic resistance to currently recommended first‐line ART in the UK. Very little relevant resistance was observed to currently recommended first‐line NRTIs or PIs: 1.2% and 0.9%, respectively. In particular, the frequently detected TAMs are no longer of direct clinical relevance in light of the replacement of zidovudine by tenofovir in first‐line therapy. A higher incidence of predicted phenotypic resistance was seen to first‐line NNRTIs, with 8.2% of individuals having predicted low‐, intermediate‐ or high‐level resistance to current drugs in this class; 6.2% of individuals had predicted resistance to rilpivirine and 3.4% to efavirenz (Fig. 2). Resistance to rilpivirine was largely low‐level resistance and probably a consequence of cross‐resistance to other NNRTIs, given the limited use of the drug in this period.

**Table 3:** Comparison of transmitted drug resistance mutations (TDRMs) and predicted phenotypic resistance (Stanford scores) by drug class, 2010–2013

ART drug class	Surveillance mutations	Predicted phenotypic resistance to currently recommended first‐line ART		
	% any TDRM	% any low level/intermediate/high level	% any intermediate/high level	% any high level
Any class	7.5	9.6	3.9	2.8
NRTI	3.5	1.2	0.8	0.6
NNRTI	3.3	8.2	3.5	2.6
PI	1.7	0.9	0.2	0.1

ART, antiretroviral therapy; NRTI, nucleoside reverse transcriptase inhibitor; NNRTI, nonnucleoside reverse transcriptase inhibitor; PI, protease inhibitor.

The results of a multivariate logistic regression model of potential predictive factors for TDRMs are shown in Table 4. Although a test for interaction between year of resistance test and transmission group did not reach conventional statistical significance (*P* = 0.65), the interaction term was retained because of the strong clinical plausibility of this effect. In an additional model which excluded this interaction term, the overall odds ratios (ORs) for heterosexual men and heterosexual women versus MSM were 0.78 [95% confidence interval (CI) 0.64–0.95] and 0.79 (95% CI 0.65–0.97), respectively. The only significant predictor of TDRMs was geographical region of attending treatment centre in the UK. Compared with individuals attending London clinics, the odds of having detectable TDRMs was lower in individuals attending treatment centres in Northern Ireland, Wales, the Midlands and East of England and the South of England (52%, 49%, 40% and 20% lower, respectively). This association persisted in a sensitivity analysis limited to individuals with subtype B infection (*P* < 0.001). Neither baseline CD4 cell count nor recency of infection defined by RITA was associated with risk of TDRMs.

**Table 4:** Logistic regression model of risk factors for transmitted drug resistance mutations (TDRMs), 2010–2013

Patient characteristics	Total	TDRMs [*n* (%)]	Univariate OR	Multivariate OR	95% CI	*P*‐value
MSM						
2010	1977	194 (9.8)	Ref	Ref	–	0.06
2011	2022	183 (9.1)	0.91	0.90	0.73–1.11	
2012	2057	166 (8.1)	0.81	0.79	0.63–0.98	
2013	1477	111 (7.5)	0.75	0.73	0.56–0.94	
Heterosexual male						
2010	826	58 (7.0)	Ref	Ref	–	0.51
2011	809	44 (5.4)	0.76	0.73	0.49–1.10	
2012	618	38 (6.1)	0.87	0.83	0.54–1.27	
2013	580	38 (6.6)	0.93	0.89	0.57–1.36	
Heterosexual female						
2010	1145	75 (6.6)	Ref	Ref	–	0.86
2011	1018	68 (6.7)	1.02	1.00	0.71–1.41	
2012	825	53 (6.4)	0.98	0.95	0.66–1.38	
2013	647	38 (5.9)	0.89	0.85	0.56–1.28	
Ethnicity						
White	8169	682 (8.3)	Ref	Ref		0.16
Black African	3932	250 (6.4)	0.75	0.86	0.71–1.06	
Black Caribbean	565	35 (6.2)	0.72	0.78	0.54–1.12	
Asian	720	50 (6.9)	0.82	0.83	0.61–1.12	
Other	1139	107 (9.4)	1.14	1.12	0.90–1.40	
Age at diagnosis						
15–24 years	1907	137 (7.2)	0.85	0.82	0.68–0.99	0.07
25–39 years	7232	603 (8.3)	Ref	Ref	–	
40–49 years	3307	234 (7.1)	0.84	0.86	0.73–1.00	
≥50 years	1757	119 (6.8)	0.80	0.85	0.70–1.05	
Region						
London	7493	640 (8.5)	Ref	Ref	–	<0.01^a^
South England	2175	150 (6.9)	0.79	0.80	0.66–0.98	
North England	2237	196 (8.8)	1.03	1.02	0.84–1.22	
Midlands and East England	3208	165 (5.1)	0.58	0.60	0.49–0.73	
Wales	441	20 (4.5)	0.51	0.51	0.32–0.81	
Scotland	435	34 (7.8)	0.91	0.86	0.59–1.25	
Northern Ireland	252	11 (4.4)	0.49	0.48	0.26–0.89	
Baseline CD4 count						
<200 cells/mL	3249	235 (7.2)	0.88	1.00	0.82–1.23	0.8
200–349 cells/mL	2508	178 (7.1)	0.86	0.91	0.74–1.12	
350–499 cells/mL	2528	206 (8.1)	Ref	Ref	–	
≥500 cells/mL	3342	278 (8.3)	1.02	0.98	0.81–1.26	
Baseline VL						
<4.0 log_10_ copies/mL	1262	111 (8.8)	1.09	1.13	0.88–1.45	0.6
4.0 to <5.0 log_10_ copies/mL	2396	195 (8.1)	Ref	Ref	–	
≥5.0 log_10_ copies/mL	2268	185 (8.2)	1.00	1.02	0.82–1.26	
RITA						
Nonrecent	4864	358 (7.4)	Ref	–	–	
Recent	1098	94 (8.6)	1.18	–	–	

CI, confidence interval; MSM, men who have sex with men; OR, odds ratio; RITA, Recent Infection Testing Algorithm; VL, viral load.

a Injecting drug users and other transmission groups are included in the multivariate model but not presented here.

Of the integrase tests reported to the UKHDRD, 101 were performed in ART‐naïve individuals between January 2007 and December 2013. No major mutations were identified in these 101 individuals. Six individuals had minor or accessory mutations: one had L74M, two had V151I and three had E157Q. In isolation, these mutations are not reported to affect susceptibility to integrase inhibitors with the exception of E157Q, which confers low‐level resistance to raltegravir and elvitegravir.

## Discussion

Analysis of TDR in the ART‐naïve UK population has revealed a trend for decreasing prevalence of TDRMs from 2005 to 2013, with 7.5% of individuals tested in recent years (2010–2013) having detectable TDRM. Other European cohort studies have also recently reported temporal trends for decreasing TDR and clinically relevant resistance to first‐line treatments, including Spanish cohorts 23, 24 and the PRIMO cohort in France 16.

In the UK, the prevalence of TDRMs remains higher in MSM than in heterosexuals, consistent with other studies in high‐income countries 16, 24, 25. This difference has been consistently reported in the UK 5 and can be attributed to historical patterns of HIV acquisition. While most MSM diagnosed in the UK have acquired their infection in the UK, where access to treatment has been free for decades, most heterosexuals historically acquired HIV abroad, where resistant HIV was essentially absent as a consequence of poor availability of ART 5. However, our analyses reveal that the difference in the prevalence of TDRMs between these groups of individuals is narrowing as different temporal patterns continue to be seen in the two populations. As the proportion of heterosexual infections acquired in the UK has increased from 36% in 2005 to 59% in 2013 22, the prevalence of TDRMs in heterosexual men and women has increased since 2006 and now appears to have stabilized between 2010 and 2013. In contrast, there has been a significant decrease in detectable TDRMs among MSM, driven by declining resistance to the NRTI drug class. Resistance to NRTIs is higher in this group particularly as a consequence of historical exposure to the thymidine analogues stavudine and zidovudine, with mutations selected by these drugs known to persist for long periods of time 26 and to be onwardly transmitted from ART‐naïve individuals 27. This report found a decline in these mutations among MSM in recent years, which suggests that they confer a slight fitness disadvantage, and a continued decline in their frequency is anticipated.

The prevalence of TDRMs in recent years, 7.5%, is a little lower than has been reported by other European cohort studies for the years since 2010 16, 23, 24, 28, 29. The incidence of dual or triple class resistance in ART‐naïve individuals is reassuringly very low. The TDRMs detected have a minimal effect at a population level on currently recommended first‐line treatment options in the UK, with predicted phenotypic resistance to individual PIs and NRTIs generally being <1%. In contrast, the prevalence of predicted NNRTI resistance was higher; 6.2% and 3.4% of individuals had resistance to rilpivirine and efavirenz, respectively. Resistance testing prior to ART initiation is therefore particularly useful if patients are to be started on an NNRTI‐containing first‐line regimen to ensure that the virus is fully susceptible to the chosen third agent. The K65R mutation, which confers resistance to tenofovir, was rarely detected as a transmitted mutation, supporting previous work by our group suggesting that this should not affect the success of tenofovir‐containing pre‐exposure prophylaxis (PreP) if its use in the UK becomes more widespread 30.

Our analysis found that region of treatment centre was a significant predictor for the presence of TDRMs in the UK population. Individuals referred for resistance tests from treatment centres in Northern Ireland, Wales, the Midlands and East England and the South of England were less likely to have detectable TDRMs than those referred from London centres. The cause of this is difficult to determine and will be influenced by the extent to which transmission networks are regional or national. Recent work has shown that a high proportion of drug‐resistant HIV transmission in the UK is from ART‐naïve individuals 27, 31. It is possible, therefore, that current geographical variation reflects historical differences in the original emergence of resistant strains, for example, as a result of earlier access to ART drugs or the use of different ART in London. A related explanation is that the average interval between HIV infection and onward transmission could be shorter in London than in other parts of the UK, thereby increasing the chance of onward transmission of resistant virus (rather than the outgrowth of wild‐type virus).

The interpretation of time trends in TDRM prevalence could be affected by temporal changes in the timing of HIV diagnoses as there is evidence for earlier detection of infections in more recent calendar years. We did not observe an association between markers of recent infection, either high CD4 count or a low avidity index, and prevalence of TDRMs. This might appear to be a surprising finding as resistant viruses in patients with long‐standing infection tend to be outcompeted by wild‐type viruses and cease to become detectable 26. However, this effect could be masked by changes in TDRM prevalence by year of infection. It is a limitation of our analysis that baseline CD4 count was missing for 29% of individuals. Baseline HIV‐1 viral load measurements were not available for a large proportion of individuals (64%), making meaningful interpretation of these data difficult.

As integrase resistance testing is not currently recommended in ART‐naïve individuals in the UK, the number of tests available to detect TDRMs conferring resistance to integrase inhibitors was small and possibly selective. No major resistance‐conferring mutations were identified in the 101 individuals analysed. However, with increasing use of integrase inhibitors in the UK, and a move to use them in first‐line regimens, it seems important to monitor TDR to this drug class 32. Some form of sentinel surveillance may be more cost‐effective than universal testing while the prevalence of TDRMs remains low.

In conclusion, our data demonstrate an overall decrease in the prevalence of TDRMs in the UK, although differences were evident between MSM and those living with heterosexually acquired HIV infection. There remains some risk of first‐line therapy with efavirenz or rilpivirine being compromised by the detected TDRMs, but clinically relevant resistance to NRTIs, PIs and integrase inhibitors is rarely seen. We recommend implementing some sentinel surveillance, at selected treatment centres, for TDRMs conferring resistance to integrase inhibitors, given their increasing use as a first‐line treatment in the UK.

## Funding

This work was supported by the UK Medical Research Council (Award Number 164587).

## The UK HIV Drug Resistance Database

Steering Committee: Celia Aitken (Gartnavel General Hospital, Glasgow); David Asboe, Anton Pozniak (Chelsea & Westminster Hospital, London); Patricia Cane (Public Health England, Porton Down); David Chadwick (South Tees Hospitals NHS Trust, Middlesbrough); Duncan Churchill (Brighton and Sussex University Hospitals NHS Trust); Duncan Clark (St Bartholomew's and The London NHS Trust); Simon Collins (HIV i‐Base, London); Valerie Delpech (Centre for Infections, Public Health England); Samuel Douthwaite (Guy's and St Thomas’ NHS Foundation Trust, London); David Dunn, Esther Fearnhill, Kholoud Porter, Anna Tostevin, Ellen White (MRC Clinical Trials Unit at UCL, London); Christophe Fraser (Imperial College London); Anna Maria Geretti (Institute of Infection and Global Health, University of Liverpool); Antony Hale (Leeds Teaching Hospitals NHS Trust); Stéphane Hué (London School of Hygiene and Tropical Medicine, London); Steve Kaye (Imperial College, London); Paul Kellam (Wellcome Trust Sanger Institute & University College London Medical School); Linda Lazarus (Expert Advisory Group on AIDS Secretariat, Public Health England); Andrew Leigh‐Brown (University of Edinburgh); Tamyo Mbisa (Virus Reference Department, Public Health England); Nicola Mackie (Imperial NHS Trust, London); Samuel Moses (King's College Hospital, London); Chloe Orkin (St. Bartholomew's Hospital, London); Eleni Nastouli, Deenan Pillay, Andrew Phillips, Caroline Sabin (University College London Medical School, London); Erasmus Smit (Public Health England, Birmingham Heartlands Hospital); Kate Templeton (Royal Infirmary of Edinburgh); Peter Tilston (Manchester Royal Infirmary); Ian Williams (Mortimer Market Centre, London); Hongyi Zhang (Addenbrooke's Hospital, Cambridge).

Coordinating Centre: MRC Clinical Trials Unit at UCL (David Dunn, Keith Fairbrother, Esther Fearnhill, Kholoud Porter, Anna Tostevin and Ellen White).

Centres contributing data: Clinical Microbiology and Public Health Laboratory, Addenbrooke's Hospital, Cambridge (Jane Greatorex); Guy's and St Thomas’ NHS Foundation Trust, London (Siobhan O'Shea, Jane Mullen); PHE – Public Health Laboratory, Birmingham Heartlands Hospital, Birmingham (Erasmus Smit); PHE – Virus Reference Department, London (Tamyo Mbisa); Imperial College Health NHS Trust, London (Alison Cox); King's College Hospital, London (Richard Tandy); Medical Microbiology Laboratory, Leeds Teaching Hospitals NHS Trust (Tracy Fawcett); Specialist Virology Centre, Liverpool (Mark Hopkins, Lynne Ashton); Department of Clinical Virology, Manchester Royal Infirmary, Manchester (Peter Tilston); Department of Virology, Royal Free Hospital, London (Clare Booth, Ana Garcia‐Diaz); Edinburgh Specialist Virology Centre, Royal Infirmary of Edinburgh (Jill Shepherd); Department of Infection & Tropical Medicine, Royal Victoria Infirmary, Newcastle (Matthias L. Schmid, Brendan Payne); South Tees Hospitals NHS Trust, Middlesbrough (David Chadwick); Department of Virology, St Bartholomew's and The London NHS Trust (Spiro Pereira, Jonathan Hubb); Molecular Diagnostic Unit, Imperial College, London (Steve Kaye); University College London Hospitals (Stuart Kirk); West of Scotland Specialist Virology Laboratory, Gartnavel, Glasgow (Rory Gunson, Amanda Bradley‐Stewart, Celia Aitken).
